# Research on the Design of Micromixer Based on Acoustic Streaming-Driven Sharp-Edge Structures

**DOI:** 10.3390/s25226886

**Published:** 2025-11-11

**Authors:** Kaihao Bai, Heting Qiao, Jixiang Cai, Jinlong Hu, Zhiqi Wang

**Affiliations:** School of Mechanical Engineering, Inner Mongolia University of Technology, Hohhot 010051, China; 18353874695@163.com (K.B.); qiaoheting311@163.com (H.Q.); jxcai@imut.edu.cn (J.C.); 17647608615@163.com (J.H.)

**Keywords:** acoustic streaming, micromixer, sharp-edge structures, mixing efficiency

## Abstract

This paper presents a three-dimensional, acoustic streaming-driven circular micromixer with sharp-edge structures and the coupling mechanism between acoustic streaming and background flow in biological systems. A piezoelectric transducer induces vibrations in the sharp-edge structures, generating a localized, intense acoustic field that produces a nonlinear acoustic streaming vortex at the tip. The disk-shaped mixing chamber design enhances acoustic field perturbation. This study incorporates the actual background flow field into the model to elucidate the strong interaction between acoustic streaming and steady-state flow. In the sharp-edge structural region, structural curvature induces local variations in acoustic amplitude, generating a non-zero mean Reynolds stress that significantly perturbs the background laminar flow, reduces flow stability, and substantially enhances mixing. The effects of displacement amplitude, Reynolds number, sharp-edge angle, and excitation frequency on the mixing efficiency are systematically investigated. Furthermore, the mixing performances of two different fluids, water and blood, are compared to elucidate the influence of fluid properties on mixing behavior. This mechanism provides theoretical support for microscale active mixing and offers novel insights for microfluidic device design.

## 1. Introduction

Microfluidics, also known as micro total analysis systems or lab-on-a-chip, has garnered significant interest in scientific studies since its inception in the 1990s [1]. With advancements in microprocessing, researchers and scholars have included micropumps, microvalves, and various functioning components into microfluidic chips, which occupy a space of merely a few square centimeters. Microfluidic chips have gained extensive application in biomedicine [2], chemical synthesis [3], drug screening [4], DNA sequencing [5], and other domains due to their miniaturization, portability, rapid reaction, and minimal sample consumption. A biosensor is an analytical instrument that integrates a biorecognition component with a physicochemical transducer, enabling the conversion of specific biomolecular interactions into quantifiable electrical, optical, or other physical signals. It possesses extensive applications in medical diagnostics, environmental monitoring, food safety, drug discovery, and various other domains. A miniaturized and portable sensing device is crucial in biomedicine for sample preparation, drug delivery, and chemical and biochemical processes [6]. The micromixer and biosensors work together to improve the sensitivity and reaction time of the sensor, demonstrating their connection. In order to guarantee that the target molecules in the sample make complete contact with the sensor’s sensing surface, the micromixer maximizes liquid mixing efficiency. This speeds up reactions and increases signals. By carefully regulating fluid flow and encouraging interactions between biomolecules and sensing materials, a micromixer used in microfluidic systems can increase detection precision and reaction time. Because it improves the detection of low-concentration chemicals, this integration is especially crucial for portable biosensors.

In the micromixer, the Reynolds number is low, rendering the inertial effects negligible due to the slow fluid flow [1,7]. Under these conditions, the microscale mixing process primarily depends on molecular diffusion, resulting in a marked decrease in mixing efficiency and an extended duration, which constrains biological applications [8]. The mixing efficiency of samples and reagents in biosensors is constrained. To tackle this technical difficulty, two categories of mixing devices are presently proposed: passive micromixing devices and active micromixing devices. Passive micromixing devices primarily depend on molecular diffusion and the modulation of chaotic advection for mixing. The molecular diffusion process is typically accomplished by enlarging the characteristic length or width of the microfluidic channel to enhance the contact area and reaction time, whereas chaotic advection is attained by introducing obstacles or grooves within the microchannel to complicate its geometry [9,10]. The efficacy of passive micromixing devices is significantly influenced by the micromixer’s design and can be modified just by altering a restricted set of parameters (e.g., flow rate) to control the reaction time post-system configuration. This leads to passive micromixing devices possessing extended mixing channels or intricate shapes. Active micromixing devices offer a regulated mixing process, eliminate intricate flow path architectures, and achieve faster mixing times than passive mixing devices. The application of external energy fields, including magnetic [11], electric [12], and acoustic fields [13], improves inter-fluid mixing and markedly decreases the mixing cycle duration. Consequently, micromixing devices utilizing acoustic field regulation have garnered significant attention owing to their distinctive physical characteristics. In comparison to alternative driving systems, acoustofluidic technology offers notable advantages, including non-invasive manipulation (as opposed to electric fields), superior energy conversion efficiency, and exceptional biocompatibility.

Acoustic field micromixers are primarily classified into three categories: acoustic streaming-based sharp-edge micromixers [14], bubble-based micromixers [15], and surface acoustic wave (SAW) micromixers [16]. SAW micromixers necessitate piezoelectric materials as a substrate, hence elevating costs and complicating processing; conversely, bubble acoustic–fluid micromixers do not require external substrates but are sometimes constrained by unstable bubble formations during fluid mixing. The exceptional dependability and stability of the sharp-edge structures in acoustic streaming-based micromixers provide efficient and portable mixing [17]. The acoustic microfluidic chip featuring sharp-edge microstructures was initially created by Po-Hsun Huang, who integrated these structures into microfluidics and utilized them in several applications, including micromixing [18], material creation [19], and cell division [20]. The study employed a fluorescent particle tracer flow field methodology to examine the flow field characteristics surrounding sharp-edge microstructures. It was discovered that under external piezoelectric vibrational excitation, stable vortex phenomena emerged near the sharp microstructures, which were associated with the stable acoustic streaming generated by these vortices [18]. Doinikov et al. [21] formulated an analytical theory to evaluate the acoustic velocity field produced at the apex of the wedge-shaped structure, and simulations indicate that the acoustic current is constituted by two counter-rotating vortices at the apex of the wedge. Nama et al. [14,22] introduced a numerical analytical technique grounded in the Nyborg ingress theory and acoustic streaming theory to model the flow field around the sharp microstructure. The findings indicate that channel dimensions substantially influence vortex and mixer efficacy owing to the nonlinearity of acoustic streaming. The effects of power, background flow velocity, and the location and geometry of sharps on the acoustic streaming field are further examined. The results indicate that increasing power enhances mixing efficiency, as the average Lagrangian velocity influences fluid mixing, thereby disturbing the background flow field. Kim et al. [23] included a micromixer into biosensors by utilizing natural convection alongside the alternating heating of two heaters to facilitate effective micromixing of influenza viral DNA. Bachman et al. [24] developed an innovative acousto-hydrodynamic micromixer to facilitate efficient mixing across a broad spectrum of flow velocities by integrating acoustic and hydrodynamic mixing, potentially enhancing the efficacy of biosensors and multistep bioanalysis. Zhang et al. [25] abandoned conventional perturbation theory and employed direct numerical simulation (DNS) to directly link the acoustic field with the flow field, therefore enhancing prediction accuracy substantially.

This study presents an acoustically driven micromixer with a sharp-edge structures design to address the shortage of specialized micromixer in biosensor applications. The main innovation lies in capturing the nonlinear acoustic streaming behavior at the sharp-edge structures, employing an entirely new geometric structure, and modeling in COMSOL Multiphysics 6.2. A multiphysics coupling model was established by integrating “pressure acoustics, frequency domain”, “thermo-viscous acoustics, frequency domain”, and “laminar flow”, directly linking the acoustic and flow fields. This coupling enables an interaction between the background flow and the vortex induced by the acoustic field. This approach reduces computational complexity and enhances laminar flow disruption through acoustic streaming, thereby greatly improving mixing efficiency. To systematically evaluate device performance, key control parameters—including displacement amplitude, Reynolds number, the angles of sharp-edge, and applied frequency—were analyzed through numerical simulations and parametric studies to identify optimal conditions. This research introduces innovative strategies for microfluidic devices, enabling active mixing in biological applications.

## 2. Mathematical Formulation and Numerical Method

### 2.1. Geometric Structure

**Figure 1:**
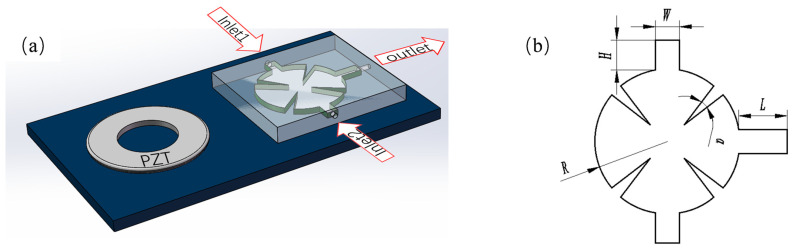
Schematic diagram of acoustic streaming micromixer: (**a**) schematic diagram of microfluidic chip; (**b**) Micromixer Geometry.

Figure 1a presents the schematic of the sharp-edge structured micromixer. A high-frequency AC voltage signal is converted into rapid, small-scale mechanical vibrations through the inverse piezoelectric effect using a piezoelectric transducer (PZT). These vibrations are transmitted to the micromixer’s sharp-edged structures, exciting the fluid inside the microchannel and generating acoustic waves. Strong vortices are formed when acoustic energy is converted into steady macroscopic flow (acoustic streaming) through nonlinear acoustic wave propagation. These vortices impose strong shear and stretching/folding stresses on the fluid, greatly enhancing fluid layer mixing. The device employs an axisymmetric flow channel design. Both inlet and outlet channels have a cross-sectional width of *W* = 1 mm, an inlet height of *H* = 1.25 mm, an outlet length of *L* = 2 mm, and the entire mixing chamber has a thickness of 0.5 mm. The circular mixing chamber has a radius of *R* = 3 mm and contains four sharply angled cells. These cells are positioned at 45° and 135° within the chamber, each with an apex angle of *α* = 15° (Figure 1b).

**Figure 2:**
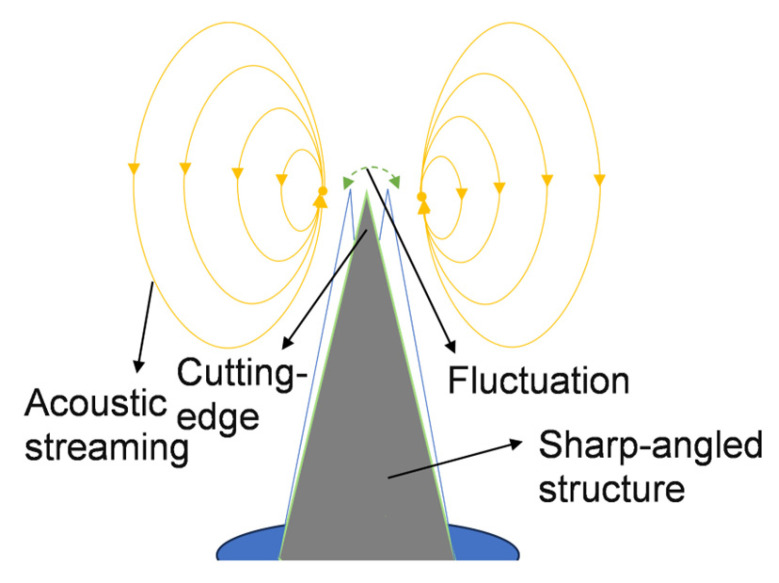
Schematic diagram of acoustic streaming generation by a sharp-edge structure.

When the sharp-edge structures oscillate, a pair of counter-rotating vortices emerges around the apex [10] (Figure 2). This acoustic streaming effect disturbs the laminar flow interface, thereby enhancing fluid mixing. Various theoretical approaches have been employed to account for differences in tip geometry and fluid configuration. Key parameters—including tip displacement amplitude, Reynolds number, the angles of sharp-edge, and applied frequency—have been optimized to elucidate the working principle and achieve efficient mixing.

### 2.2. Mathematical Formulation

The principle of acoustic streaming underpins the micromixing mechanism in the angles of a sharp-edge micromixer. Traditional theories of acoustic streaming neglect the influence of the background flow field, assuming it remains stationary and accounting only for the motion induced by the acoustic field. In this study, an external flow field within the microchannel is incorporated, interacting with the acoustic field.

#### 2.2.1. Fluid Flow

In the microfluidic chip, the flow characteristics of a fluid are commonly described by the Reynolds number (*Re*), which is defined as the ratio of inertial forces to viscous forces:

(1) $$Re=\frac{\rho u_{t}L}{\mu }$$

where *ρ* is the fluid density, *u_t_* is the characteristic velocity at the inlet of the flow field, *L* is the characteristic length of the flow field, and *μ* is the hydrodynamic viscosity. The *Re* affects the stability and type of fluid flow. *Re* < 2100 in the flow field for the laminar state, when the viscous force dominates; *Re* > 4000 for the turbulent state, when the inertial force dominates; and *Re* between 2100~4000 for the transition flow state. In the microfluidic chip, the *Re* is small, and this paper studies the low *Re* flow of incompressible fluid.

**Figure 3:**
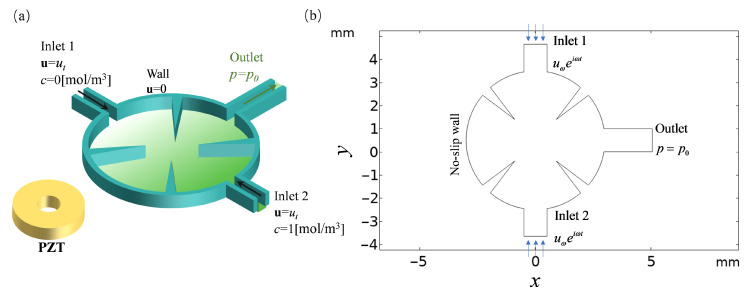
(**a**) Concentration and laminar boundary conditions in the study domain; (**b**) boundary conditions of the acoustic field in the study domain.

The governing principles of linear viscous fluid motion are the conservation of mass (continuity equation) and the conservation of momentum (Navier–Stokes equation) [26]:

(2) $$\frac{\partial \rho }{\partial t}+\rho \nabla \cdot u=0$$

(3) $$\rho \frac{\partial u}{\partial t}+\rho \left(u\cdot \nabla \right)u=-\nabla p+\mu \nabla^{2}u+\left(\mu_{b}+\frac{1}{3}\mu \right)\nabla \left(\nabla \cdot u\right)$$

where **u** is the velocity vector, *p* is the compressive stress (positive pressure), and *μ* and *μ_b_* are the kinetic and bulk viscosities, respectively. To describe the fluid flow, these equations require the equations of the intrinsic relationship between pressure and density [27,28], assuming that the fluid is positively pressurized; that is, there is a linear relationship between pressure and density, an approximation commonly used in acoustics and small amplitude perturbation analysis. This approximation applies to the behavior of compressible fluids under small perturbations and is a common assumption in the study of acoustic wave propagation and acoustic streaming. It is assumed that the pressure *p* and density *ρ* are satisfied:

(4) $$p=c_{0}^{2}\rho$$

where *c_0_* is the speed of sound in a stationary fluid and is a function of density, and the flow that is the subject of this study is considered as an incompressible flow. However, to accurately model the key physical process of acoustic wave propagation, we introduce the linear equation of state (4) to represent the compressibility effect of the fluid under acoustic wave action. The boundary conditions in the fluid flow are set to

(5) $$\left\{\begin{array}{l} u=u_{t}\;\mathrm{on}\;\Gamma_{inlet1}\\ u=u_{t}\;\mathrm{on}\;\Gamma_{inlet2}\\ u=0\;\;\mathrm{on}\;\Gamma_{wall}\\ p=p_{0}\;\mathrm{on}\;\Gamma_{\mathrm{outlet}}\; \end{array}\right.$$

where Γ*_inlet_*_1_ and Γ*_inlet_*_2_ are the inlets of the mixer, Γ*_outlet_* is the outlet of the mixer, and Γ*_wall_* represents a no-slip wall boundary. *u_t_* is the inlet velocity of the two inlets, and the velocities of the two inlets are equal. *p*_0_ is the pressure at the outlet, set to 0 (Figure 3).

Microfluidic chips are widely used in the biomedical industry. To adapt to different application scenarios, this paper uses typical human blood (37 °C) for comparison. Blood is a non-Newtonian fluid, and the relationship between stress and strain rate does not follow the friction law mentioned above. Considering the shear thinning effect of blood, this paper chooses the modified Carreau model as the dynamic viscosity:

(6) $$\eta (\gamma_{1})=\eta_{\infty }+\left(\eta_{0}-\eta_{\infty }\right){\left(1+{\left(\lambda \gamma_{1}\right)}^{2}\right)}^{\frac{\left(n-1\right)}{2}}$$

(7) $$\gamma_{1}=\sqrt{2S:S}\;S=\frac{1}{2}\left[\nabla u+{\left(\nabla u\right)}^{T}\right]$$

where *η*(*γ*_1_) is the dynamic viscosity, *η_∞_* is the infinite shear viscosity, *η*_0_ is the zero shear viscosity, *λ* is the relaxation time, *n* is the power law exponent, *γ*_1_ is the shear rate, and ***S*** is the strain tensor. Parameter values are as follows: *η_∞_* = 0.056 Pa·s, *η*_0_ = 0.0035 Pa·s, *λ* = 3.3 s, *n* = 0.36.

#### 2.2.2. Acoustic Field

According to the ingress theory, the velocity *u*, pressure *p*, and density *ρ* of the flow field can be expanded and expressed as follows:

(8) $$u=u_{0}+\varepsilon {\tilde{u}}_{1}+\varepsilon^{2}{\tilde{u}}_{2}+\ldots +O\left(\varepsilon^{n}\right)=u_{0}+u_{1}+u_{2}+\ldots$$

(9) $$p=p_{0}+\varepsilon {\tilde{p}}_{1}+\varepsilon^{2}{\tilde{p}}_{2}+\ldots +O\left(\varepsilon^{n}\right)=p_{0}+p_{1}+p_{2}+\ldots$$

(10) $$\rho =\rho_{0}+\varepsilon {\tilde{\rho }}_{1}+\varepsilon^{2}{\tilde{\rho }}_{2}+\ldots +O\left(\varepsilon^{n}\right)=\rho_{0}+\rho_{1}+\rho_{2}+\ldots$$

where *ε* is a parameter with a small dimensionless value, *u_i_* is the ith-order velocity, i.e., **u_0_** is the zeroth-order component of the velocity (the background flow field), **u_1_** is the first-order component of the velocity, **u_2_** is the second-order component of the velocity, $\tilde{u}$ is the ith-order velocity with perturbation parameter, *p_i_* is the ith-order pressure with perturbation parameter, $\tilde{p}$ is the ith-order pressure with perturbation parameter, *ρ_i_* is the ith-order density, and $\tilde{\rho }$ is the ith-order density with perturbation parameter. Here, *ε* can be chosen to be the ratio of the amplitude of the first-order velocity to the speed of sound in a stationary fluid. Usually, the zero-order velocity field **u_0_** is assumed to be zero, but in this study, there is a background flow field in the microchannel, so Equations (4)–(6) are substituted into (1) and (2) to obtain the zero-order set of equations, i.e., the flow field without acoustic field action:

(11) $$\frac{\partial \rho_{0}}{\partial t}+\rho_{0}\left(\nabla \cdot u_{0}\right)=0$$

(12) $$\rho_{0}\frac{\partial u_{0}}{\partial t}+\rho_{0}\left(u_{0}\cdot \nabla \right)u_{0}=-\nabla p_{0}+\mu \nabla^{2}u_{0}+\left(\mu_{b}+\frac{1}{3}\mu \right)\nabla \left(\nabla \cdot u_{0}\right)$$

For a flow field exhibiting first-order acoustic field action, the first-order equations governing the acoustic response of the fluid are derived as follows [17]:

(13) $$\frac{\partial \rho_{1}}{\partial t}+\nabla \cdot \left(\rho_{0}u_{1}+\rho_{1}u_{0}\right)=0$$

(14) $$\begin{array}{c} \rho_{0}\frac{\partial u_{1}}{\partial t}+\rho_{1}\frac{\partial u_{0}}{\partial t}+\rho_{0}\left(u_{1}\cdot \nabla \right)u_{0}+\rho_{0}\left(u_{0}\cdot \nabla \right)u_{1}+\rho_{1}\left(u_{0}\cdot \nabla \right)u_{0}\\ =-\nabla p_{1}+\mu \nabla^{2}u_{1}+\left(\mu_{b}+\frac{1}{3}\mu \right)\nabla (\nabla \cdot u_{1}) \end{array}$$

For a flow field exhibiting second-order acoustic field action, the equations governing the second-order acoustic response of the fluid are derived as follows:

(15) $$\langle \frac{\partial \rho_{2}}{\partial t}\rangle +\nabla \cdot \left(\langle \rho_{0}u_{2}\rangle +\langle \rho_{2}u_{0}\rangle \right)=-\nabla \cdot \langle \rho_{1}u_{1}\rangle$$

(16) $$\begin{array}{c} \langle \rho_{0}\frac{\partial u_{2}}{\partial t}\rangle +\langle \rho_{2}\frac{\partial u_{0}}{\partial t}\rangle +\langle \rho_{1}\frac{\partial u_{1}}{\partial t}\rangle +\langle \rho_{0}\left(u_{1}\cdot \nabla \right)u\rangle +\langle \rho_{0}\left(u_{0}\cdot \nabla \right)u_{2}\rangle +\\ \langle \rho_{0}\left(u_{2}\cdot \nabla \right)u_{0}\rangle +\langle \rho_{1}\left(u_{0}\cdot \nabla \right)u_{1}\rangle +\langle \rho_{1}\left(u_{1}\cdot \nabla \right)u_{0}\rangle +\langle \rho_{2}\left(u_{0}\cdot \nabla \right)u_{0}\rangle \\ =-\nabla \langle p_{2}\rangle +\mu \nabla^{2}\langle u_{2}\rangle +\left(\mu_{b}+\frac{1}{3}\mu \right)\nabla \langle \nabla \cdot \langle u_{2}\rangle \rangle \end{array}$$

where <A> represents the temporal average of A across the vibration time interval. The inertia term in Equation (14) is substantial and must be included in the formulation. To comprehensively address the viscous attenuation effects of acoustic waves within and beyond the boundary layer, the final term in Equation (14), associated with volumetric viscosity, must be preserved [29]. Equations (11)–(14) demonstrate that the acoustic streaming produced by the sharp-edge structures results from the combined effects of the background flow field, the first-order acoustic field, and the second-order acoustic field, with both the first-order and second-order acoustic fields being affected by the background flow field.

In the context of first-order boundary conditions, the exterior structure conveys the auditory flow into the mixer interior via the vibratory displacement of the sharp-edge structures, with the equation governing the driving displacement of this structure being

(17) $$u_{1}=\left[\begin{array}{l} u_{1}\\ v_{1}\\ w_{1} \end{array}\right]=\left[\begin{array}{c} 2\pi f_{0}d_{0}\\ 2\pi f_{0}d_{0}\\ 0 \end{array}\right]$$

where *f*_0_ is the drive frequency and *d*_0_ is the displacement amplitude of the sharp-edge structures (Figure 3).

#### 2.2.3. Material Transport

According to the diffusion theory, the micromixing of the liquid inside the microfluidic channel satisfies the convection-diffusion equation:

(18) $$\frac{\partial c}{\partial t}+\nabla \cdot \left(cu\right)=D\nabla^{2}c$$

where *c* is the concentration and *D* is the diffusion coefficient. The boundary conditions set in substance transport are (Figure 3) as follows:

(19) $$\left\{\begin{array}{l} c=0\;\mathrm{mol}/m^{3}\;\;\;on\;\Gamma_{Inlet1}\\ c=1\;\mathrm{mol}/m^{3}\;\;\;on\;\Gamma_{inlet2}\\ cu=0\;\;\;\;\;\;\;\;\;\;\;\;\;\;on\;\Gamma_{wall} \end{array}\right.$$

Table 1 lists the parameter values used in this study, including fluid density, sound velocity, viscosity, etc.

### 2.3. Acoustic–Fluid Coupling Mechanism

#### 2.3.1. Principle of Acoustic–Fluid Coupling

Although the physical processes that take place close to sharp-edge structures (on the micrometer scale) are far smaller than the acoustic waves’ wavelength (on the millimeter scale), acoustic waves are compressible waveforms. Acoustic waves are therefore taken to be incompressible waveforms for the sake of this investigation. In classical perturbation theory, the overall velocity field and pressure field are decomposed into two parts, as the core of acoustic streaming comprises both fast oscillations and steady-state fluid flow. But since there is a background flow field in this study, the whole velocity field is broken down into three parts (Figure 3): the acoustic streaming velocity **u_s_** (**u_2_**), the acoustic oscillation velocity **u_ω_** (**u_1_**), and the background flow field **u_0_**. Piezoelectric sensors create fluid vibrations, which in turn cause the acoustic oscillation velocity [30,31,32]:

(20) u=u0+Re(uωeiωt)+usp=p0+Re(pωeiωt)+ps

The fluid’s oscillatory motion at the acoustic frequency *ω* is described by the complex amplitudes **u_ω_** and *p_ω_*, of which Re(·) represents the real portion. A body force *F_s_* (average Reynolds stress), which is the source term driving the acoustic streaming and represents the time-averaged effect of the acoustic momentum flux, is produced on the acoustic field as a result of the interaction of the nonlinear effects of the acoustic field itself (inertial terms):

(21) Fs=−ρ2Re(uω⋅∇)uωT

where T represents conjugation. Substituting Equation (21) into the NS equation, we obtain

(22) ∂∂tRe(uωeiωt)⋅∇+[us+u0+Re(uωeiωt)]⋅∇us+u0+Re(uωeiωt)=−1ρ∇[ps+Re(pωeiωt)]+μ∇2[us+u0+Re(uωeiωt)]

The instantaneous dynamics of the whole flow field are described by this equation. Re (**u_ω_***e^iωt^*) is not just a uniform oscillation; it is a field that changes considerably in space. This indicates that the acoustic streaming is mostly driven by the nonlinear elements in the equation, which intensify close to sharp-edge structures.

As mentioned above, in this study, the velocity field and pressure field are divided into three parts, as shown in Equation (20). Then, the momentum Equation (22) can be divided into two parts: the oscillation term (23) and the steady-state term (24):

(23) $$i\omega u_{\omega }+\left(\left(u_{s}+u_{0}\right)\cdot \nabla \right)u_{\omega }+\left(u_{\omega }\cdot \nabla \right)\left(u_{s}+u_{0}\right)=-\frac{1}{\rho }\nabla p_{\omega }+\mu \nabla^{2}u_{\omega }$$

(24) $$\left(\left(u_{s}+u_{0}\right)\cdot \nabla \right)\left(u_{s}+u_{0}\right)=-\frac{1}{\rho }\nabla p_{s}+\mu \nabla^{2}\left(u_{s}+u_{0}\right)+F_{s}$$

Equation (22) is a first-order equation used in perturbation theory to solve for the acoustic field. It clearly shows that the distribution of the acoustic field **u_ω_** and *p_ω_* is inversely affected by the steady-state flow field (**u_s_** + **u_0_**) (convection term). Equation (23) accurately describes the generation of the steady-state acoustic streaming **u_s_**.

#### 2.3.2. Numerical Modeling of Acoustic Streaming

**Figure 4:**
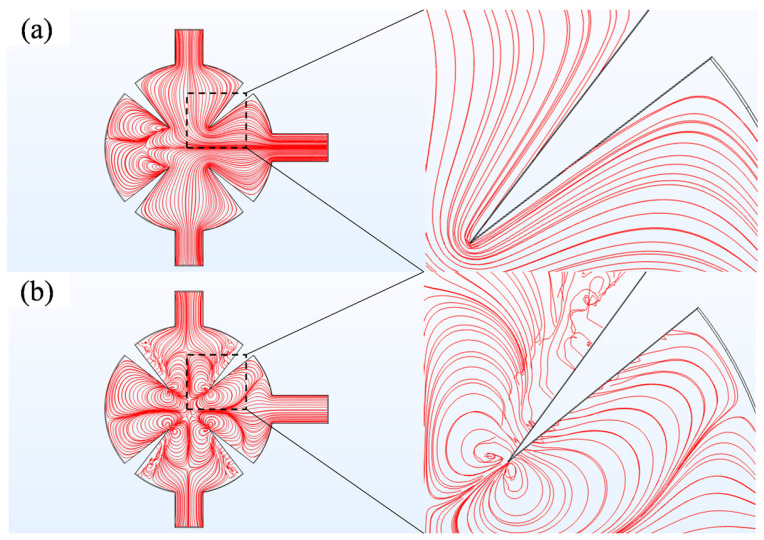
(**a**) Streamlines at no acoustic field; (**b**) acoustic field streamlines at *Re* = 0.1, *f*_0_ = 5.5 kHz, and *d*_0_ = 5 μm.

**Figure 5:**
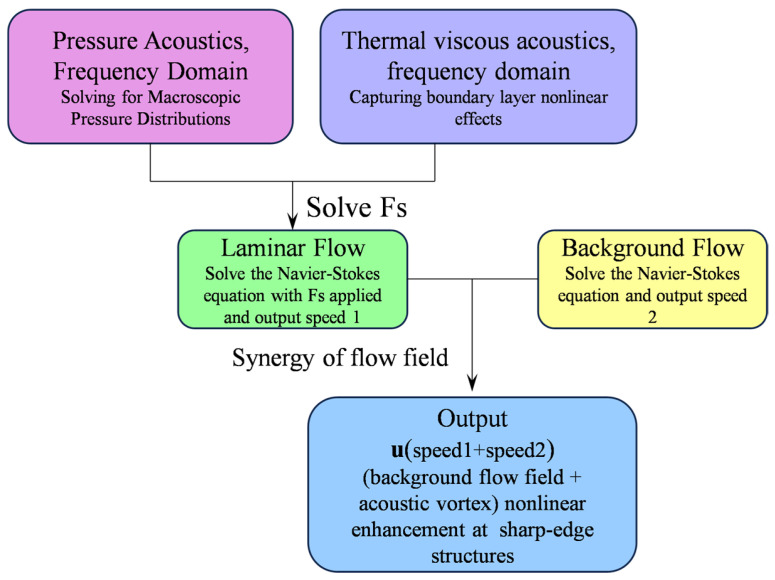
Flow chart of acoustic field coupling.

The acoustic pressure field, thermo-viscous dissipation, and fluid dynamics modules are integrated through a multiphysics coupling approach. The acoustic pressure field module solves the first-order acoustic field in the frequency domain. The fluid dynamics module computes the second-order acoustic field, while the thermo-viscous dissipation module analyzes viscous and thermal effects within the boundary layer in the frequency domain. The thermo-viscous dissipation method, combined with local mesh optimization, was employed to capture nonlinear boundary-layer effects in the sharp-edge structures region. Multiphysics coupling underpins the generation mechanism of the second-order acoustic field. The acoustic pressure field is coupled with the thermo-viscous dissipation module to calculate the average Reynolds stress, which is subsequently introduced as a source term into the fluid dynamics module. Strong interactions between acoustic streaming vortices and the background flow occur in the sharp-edge structures region, effectively enhancing mixing efficiency. To visualize the perturbation of the acoustic field on the flow field, Figure 4. compares streamline distributions under two conditions: without an acoustic field (a) and with an acoustic field (b). When the acoustic field is absent, streamlines remain smooth, representing typical laminar flow. Once the acoustic field is applied, a pair of reverse vortices emerge at the angles of the sharp edge, completely breaking the laminar interface. This is the core physical process underlying acoustic streaming-driven mixing (Figure 5).

### 2.4. Mixing Efficiency

To assess the fluid mixing within the mixer, the mixing quality (*MQ*) is evaluated using the mixing efficiency at the outlet boundary, which measures the mixing concentration index at the outlet [33]:

(25) $$c_{norm}=\frac{c-c_{min}}{c_{max}-c_{min}}$$

(26) $$\begin{array}{c} MQ=(1-\frac{\sigma }{\sigma_{\max}})\\ \sigma =\sqrt{\int_{\Gamma_{Outlet}}{\left(c-\bar{c}\right)}^{2}d\Gamma },\sigma_{\max}=\sqrt{c_{m}(1-c_{m})} \end{array}$$

where *c_norm_* represents the normalized concentration, $\bar{c}$ represents the average concentration of the cross-section at the outlet, *σ* represents the standard deviation of the cross-section at the outlet, *σ_max_* denotes the maximum standard deviation when it is completely unmixed, and *c_m_* is the theoretical average concentration. Since the concentrations at the inlet are 0 and 1 mol/m^3^, respectively, *c_m_* is 0.5 mol/m^3^. *MQ* = 0 and *MQ* = 1 represent complete separation and complete mixing. Their values range from 0 to 1. The higher the *MQ* value, the better it represents the mixing of two fluids with different concentrations, and the lower the *MQ* value, the worse the mixing effect.

### 2.5. Grid Independence Test

**Figure 6:**
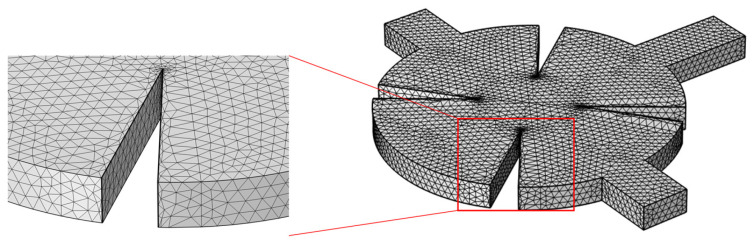
Grid structure in the micromixer.

This study employs grid convergence analysis for parameter optimization to address singularities in numerical simulations. A systematic mesh sensitivity analysis established a hierarchical meshing scheme: the maximum mesh size in the computational domain of the micromixer was set to 0.15 mm, while the minimum was 0.01 mm. Local cell constraints were applied in gradient-sensitive regions, such as the sharp-edge structure, with a maximum cell size of 0.025 mm. The final mesh was generated using unstructured free triangular elements and consisted of 103,139 cells (Figure 6) (Appendix A).

## 3. Results and Discussion

This study systematically investigates the mixing performance, fluid flow behavior, and acoustic field characteristics of an acoustofluidic micromixer based on a disk-shaped sharp-angle microstructure. The aim is to verify the feasibility and practicality of the developed microfluidic chip, providing a technical reference for its fabrication and use. Previously, the radius of curvature may have an impact on mixing, which was discussed in Appendix B. Ultimately, in conjunction with this study, the result without the radius of curvature was selected. The study covers a *Re* range of 0.1 to 10, corresponding to variations in the actual background flow rates. The angles of the sharp edges vary from 10° to 45° in terms of geometric parameters. The frequency range of the acoustic parameters is 4–16 kHz, and the displacement amplitude ranges from 1 to 5 μm. These are controlled by the frequency and voltage amplitude settings of the signal generator, respectively. In the experimental system, the signal generator produces a sinusoidal electrical signal, which is then amplified by a power amplifier. This drives a piezoelectric transducer, converting the electrical signal into mechanical vibrations at the corresponding frequency. The signal is transmitted to the microfluidic chip through coupling, exciting the sharp-angle structure and generating an acoustic field and acoustofluidic effect consistent with the simulation settings. This study evaluated the mixing efficiency (*MQ*) at the outlet through numerical simulation, and systematically analyzed key parameters, including concentration field distribution, velocity distribution, and normalized concentration curve. Additionally, the mixing characteristics of water and blood fluids under different concentration conditions were examined, providing a theoretical foundation and experimental reference for the design and operation of actual devices.

### 3.1. Effect of Displacement Amplitude

Investigation of the mixing mechanism in acoustic micromixers reveals that acoustic perturbations generated by piezoelectric transducers modulate the acoustic streaming field through the sharp-edge structures. In sharp-edge structures acoustic micromixers, the amplitude of acoustic displacement is a key parameter governing mixing efficiency, as it strongly affects acoustic streaming intensity and vortex generation. As the displacement amplitude increases, the oscillatory motion of fluid particles intensifies, producing a stronger acoustic streaming field and more complex vortex structures in the near-field region of the sharp-edge structure’s geometry. These dynamic effects destabilize the laminar boundary layer and amplify interfacial perturbations between fluid layers by inducing chaotic advection, thereby enhancing microscale mixing efficiency through interfacial interactions. Higher displacement amplitudes amplify the acoustic radiation force, generating stronger pressure gradients that enhance convective motion. Mixing efficiency is further enhanced by elevated molecular diffusion arising from substantial shear forces induced by convective motion.

**Figure 7:**
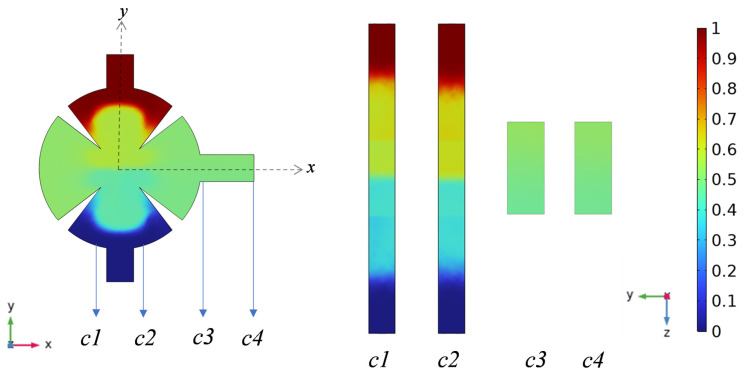
Concentration maps in xy and yz cross-sectional directions for *d*_0_ of 5 μm and *Re* of 0.1.

Figure 7 illustrates the evolution of the concentration field across four yz-sections (*c*1–*c*4) along the flow direction, under the conditions of *d*_0_ = 5 μm, *f*_0_ = 5.5 kHz, and *Re* = 0.1 when the solution is water. The cross-sections *c*1, *c*2, *c*3, and *c*4 are located at transverse coordinates of −0.7, 0.7, 3, and 5, respectively. At the inlet, the fluid exhibits laminar flow. Acoustic streaming induced by the piezoelectric transducer drives the fluid through the sharp-edge structures within the channel (sections *c*1 and *c*2). Vibrations of the sharp-edge structures disrupt the stable laminar interface, inducing flow instability and markedly enhancing inter-fluid mixing. As the fluid advances toward the outlet region (*c*3 and *c*4), the mixing process intensifies, ultimately leading to complete fluid amalgamation.

**Figure 8:**
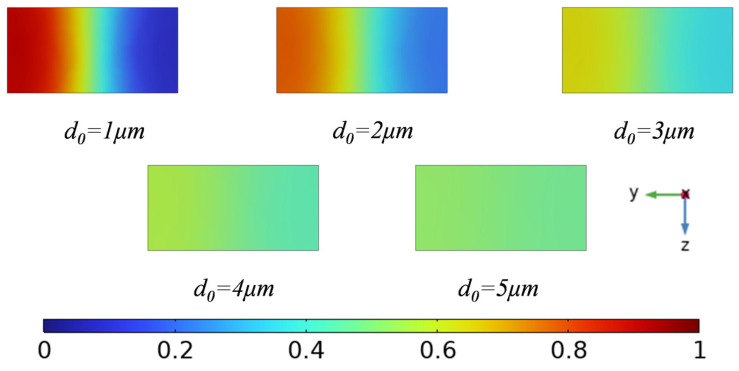
Plot of *c*4 cross-section concentration for different displacement amplitudes.

Figure 8 illustrates the cross-sectional concentration distributions at the outlet for different displacement amplitudes (*d*_0_ = 1, 2, 3, 4, 5 μm). At small displacement amplitudes, the interaction between the acoustic wave and the fluid is weak, and the acoustic radiation force is insufficient to effectively disturb the laminar interface. Consequently, the mixing process is limited, and a pronounced concentration gradient remains at the outlet. As the displacement amplitude increases, the acoustic radiation force becomes stronger, inducing significant flow perturbations and leading to a more uniform concentration distribution, thereby improving the overall mixing performance.

**Figure 9:**
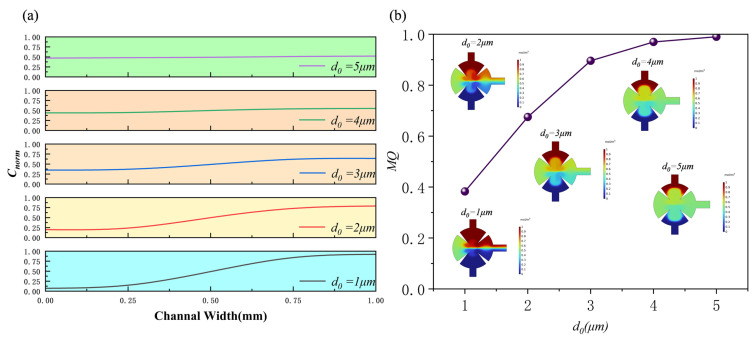
Under different displacement amplitudes: (**a**) normalized concentration profile at outlet; (**b**) mixing efficiency profile.

Figure 9 presents the normalized concentration profiles and the variation in mixing quality (*MQ*) with different displacement amplitudes. With increasing displacement amplitude, the normalized concentration curves become smoother, indicating enhanced fluid mixing. Meanwhile, the *MQ* value rises continuously, exceeding 99% when *d*_0_ = 5 μm. In addition, the vortex strength around the sharp-edge structures increases with the displacement amplitude, further promoting efficient fluid mixing within the chamber.

**Figure 10:**
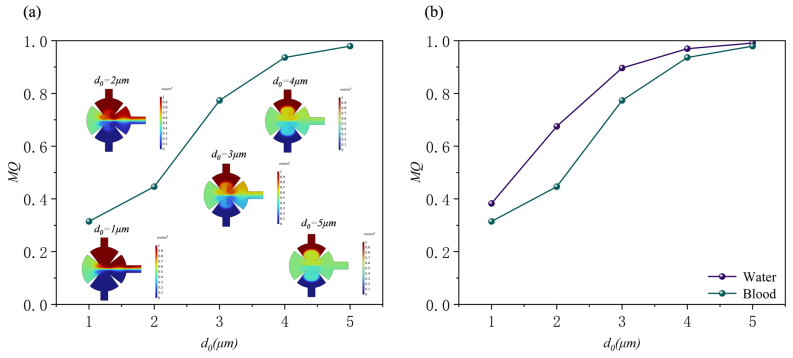
(**a**) Mixing efficiency curves at different displacement amplitudes when the fluid is human blood, (**b**) mixing efficiency curves at different displacement amplitudes when the fluid is water and human blood.

To expand its application scenarios, Figure 10 shows the concentration distribution along the yz section at the outlet for different displacement amplitudes (*d*_0_ = 1, 2, 3, 4, and 5 μm) using human blood as the solution. The results also compare the mixed concentrations of the aqueous solution and human blood at the outlet. The results show that the mixing effects of the two solutions are similar, and the degree of mixing approaches a certain level as the displacement amplitude increases.

### 3.2. Effect of Reynolds Number

**Figure 11:**
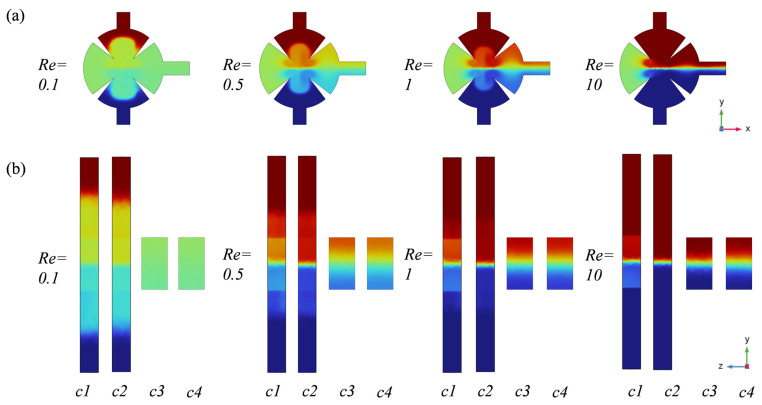
Concentration distribution of (**a**) xy cross-section and (**b**) yz cross-section at different Reynolds numbers.

In biological micromixers—such as those used for cell processing, blood fusion, and protein analysis—the Reynolds number (*Re*) typically remains below 0.1 to prevent cellular shear damage, maintain cellular activity, and ensure biomolecular stability [25]. We investigated the mixing efficiency of the micromixer at different *Re* (0.1, 0.5, 1, and 10) under the conditions of *d*_0_ = 5 μm, *f*_0_ = 5.5 kHz, and *α* = 15° when the solution is water. Figure 11. shows the concentration distribution across multiple cross-sections, displaying a symmetric profile under low *Re* conditions (*Re* = 0.1). Mixing begins closer to the inlet. This is attributed to the slow fluid flow at low *Re*, where molecular diffusion dominates and the fluid has sufficient residence time to interact with acoustic streaming around the acute corner structure, thereby enhancing mixing. Acoustic streaming generated by the interaction between the acute corner geometry and the applied acoustic field exhibits laminar flow characteristics at low *Re*, resulting in effective mixing.

As the *Re* increases to 0.5–1, a distinct concentration gradient forms at the interface in the cross-section, indicating that molecular diffusion alone is insufficient to achieve effective mixing before the fluid reaches the sharp-edge structures. The concentration distribution near the acute corner becomes asymmetric, with mixing in regions near the outlet markedly stronger than in regions farther from the outlet, contrasting with the symmetric distribution observed at low *Re*. This observation demonstrates that the sharp-edge structures’ configuration significantly affects mixing at higher flow rates. Enhanced mixing around the sharp-edge structures reflects the pronounced stretching and folding effect of the acoustic streaming generated by the sharp-edge structures on the laminar flow interface, promoting homogenization of the concentration distribution. Nonetheless, residual concentration gradients persist near the outlet. This is likely due to the short fluid residence time and the limited enhancement of mixing provided by the sharp-edge structures at this displacement amplitude.

Mixing efficiency near the sharp-edge structures decreases significantly under high-flow conditions at a *Re* of 10. This reduction is primarily attributed to the high-velocity background flow (convective effects), which weakens the acoustic streaming generated by the sharp-edge structures, regardless of any potential increase in amplitude or intensity, thereby limiting the mixing enhancement provided by acoustic streaming.

**Figure 12:**
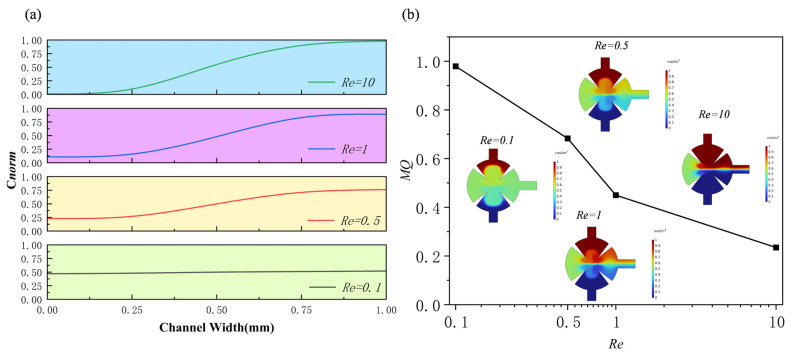
Under different Reynolds numbers: (**a**) normalized concentration profile at outlet; (**b**) mixing efficiency profile.

Figure 12 presents the variation in mixing performance under different *Re* (*Re* = 0.1, 0.5, 1, and 10). Panel (a) shows the normalized concentration distribution at the outlet, while panel (b) illustrates the variation in mixing efficiency with *Re*. The results further support the aforementioned findings: as the *Re* decreases, the acoustic radiation force and vortex-induced streaming become more pronounced, effectively disrupting the laminar interface and enhancing fluid mixing. At lower *Re* (*Re* ≤ 0.5), the concentration distribution becomes nearly uniform, and the mixing efficiency approaches 1, indicating complete mixing. In contrast, when *Re* increases to 10, the inertial effects become dominant, the acoustic streaming weakens, and the mixing efficiency decreases significantly.

**Figure 13:**
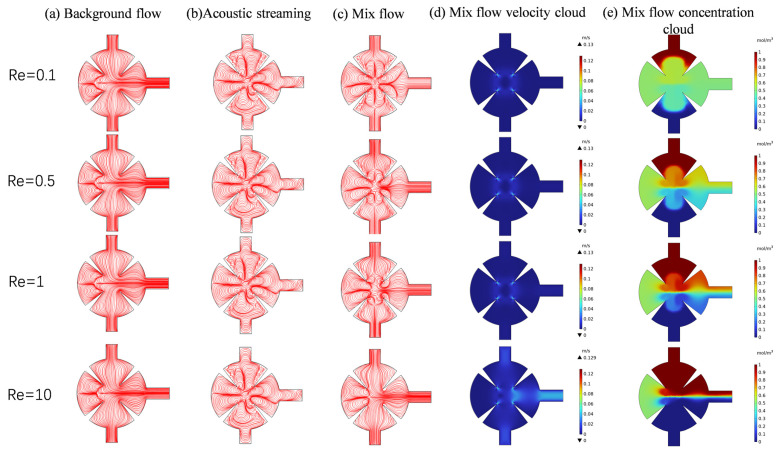
(**a**) Background flow line diagram; (**b**) acoustic streaming line diagram; (**c**) mixed flow line diagram; (**d**) mix flow velocity cloud; (**e**) mix flow concentration cloud for different Reynolds numbers.

This study investigates the velocity streamlines of background, acoustic, and combined flows at various *Re* to clarify the mixing mechanism within the microchannel. Background flow refers to channel conditions without acoustic field influence, while combined flow denotes conditions where both background and acoustic streaming coexist. Background flow and acoustic streaming are the primary factors affecting streamline distribution within the mixing cavity. Figure 13a shows that sharp-edge structures within the mixing cavity induce a localized vortex in the surrounding region, even without acoustic field influence. This vortex acts as the main driver of passive mixing within the cavity. With increasing *Re*, the vortex in the acute corner region gradually shifts toward the apex. Higher flow velocities amplify convective effects, thereby reducing mixing efficiency. Figure 13b shows that increasing *Re* causes vortices generated by acoustic streaming to gradually shift toward the tip. When background and acoustic streaming coexist (Figure 13c), the vortex mainly occupies regions near the tip, with an increase in size. At low *Re*, vortices generated by acoustic streaming merge with those from the background flow, both exhibiting the same rotational direction and magnitude. Vortices near the inlet obscure the flow, indicating that the laminar regime is disrupted by acoustic streaming upon fluid entry into the mixing chamber. As *Re* increases, flow velocity in the mixing chamber rises, acoustic streaming is suppressed by the background flow, vortices induced by the acoustic field begin migrating inward, and the influence of acoustic streaming diminishes.

**Figure 14:**
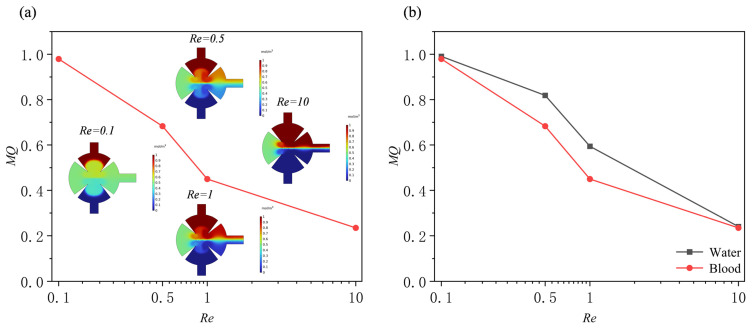
(**a**) Mixing efficiency curves at different Reynolds numbers when the fluid is human blood and (**b**) mixing efficiency curves at different Reynolds numbers when the fluid is water and human blood.

Subsequently, using human blood as a solution and, under fixed parameters (*d*_0_ = 5 μm, *f*_0_ = 5.5 kHz, α = 15°), investigated the effects of different *Re* (0.1, 0.5, 1, and 10) on the mixing efficiency of the micromixer (Figure 14a). Similar to aqueous solutions, the mixing quality of blood also showed a significant downward trend with increasing Re. However, a comparison of the two shows that the attenuation of the mixing efficiency of blood was greater than that of aqueous solutions (Figure 14b). This difference is primarily due to the shear-thinning properties of blood as a non-Newtonian fluid: at low *Re* (i.e., low shear rates), blood exhibits a high viscosity, significantly inhibiting mixing. As the Re increases (increasing shear rate), its apparent viscosity decreases, gradually approaching the viscosity level of aqueous solutions. Consequently, at higher Re, the mixing performance of the two approaches similarity.

### 3.3. Effect of the Angles of Sharp Edges

**Figure 15:**
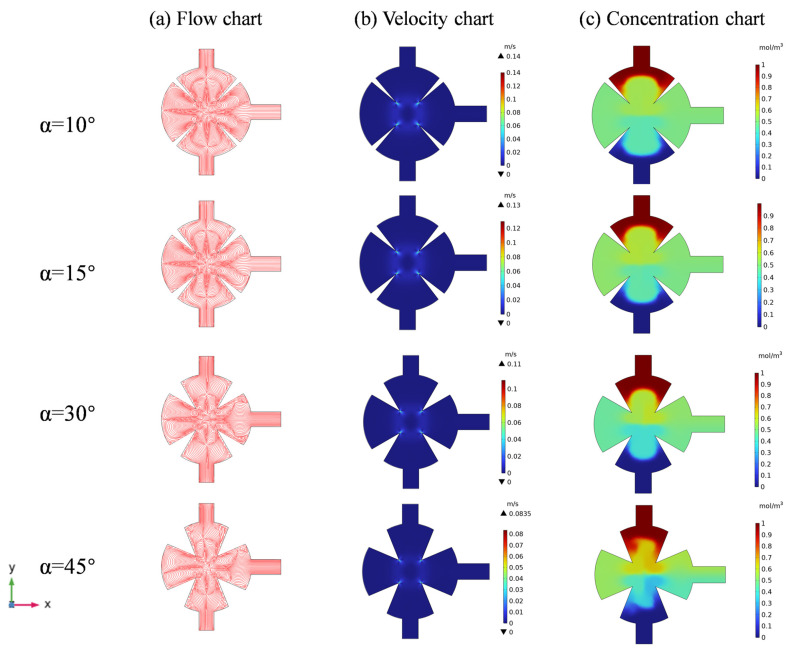
(**a**) Streamlines; (**b**) velocities; (**c**) concentrations at different angles of sharp edges.

**Figure 16:**
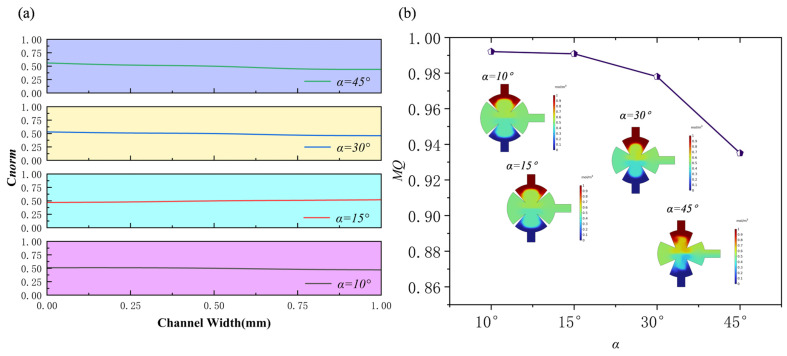
Under different angles of sharp edges: (**a**) normalized concentration profile at outlet; (**b**) mixing efficiency profile.

The angles of sharp edges are a key factor in the design of biological micromixers, including those applied in cell processing or in mixing blood with fluorescent reagents. Overly high angles of sharp edges may reduce cellular activity in the tip region. In this study, the angles of sharp edges (*α*) were evaluated at 10°, 15°, 30°, and 45° under conditions of *Re* = 1, *f*_0_ = 5.5 kHz, and *d*_0_ = 5 μm when the solution is water. The results show that increasing the angles of sharp edge affects mixing efficiency. Streamline analysis within the mixing chamber (Figure 15) shows that smaller angles of sharp edges (α) generate a broader range of acoustic streaming in the tip region. Conversely, larger angles of sharp edges (α) reduce the mixing region, as the two tips converge, compressing the vortices generated by acoustic streaming and weakening their effect. The normalized concentration curve (Figure 16a) confirms that smaller angles of sharp edges (*α*) enhance mixing and align the channel’s normalized concentration more closely with the target value of 0.5. The mixing efficiencies are 0.992 at *α* = 10°, 0.99 at *α* = 15°, 0.978 at *α* = 30°, and 0.935 at *α* = 45° (Figure 16). Although changes in the angles of sharp edges strongly affect the flow field structure and concentration distribution, the variations in overall mixing quality remain small.

**Figure 17:**
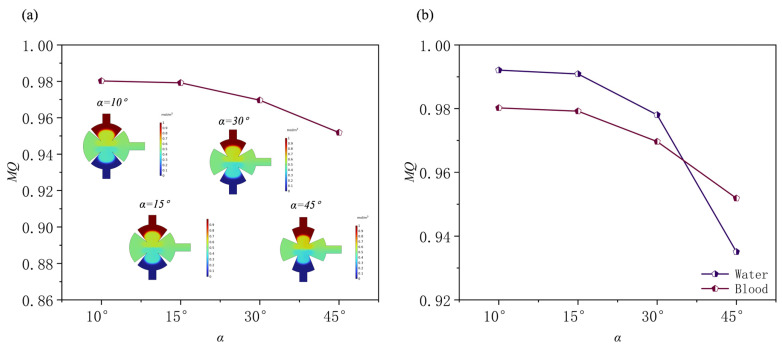
(**a**) Mixing efficiency curves at different angles of sharp edges when the fluid is human blood, and (**b**) mixing efficiency curves at different angles of sharp edges when the fluid is water and human blood.

This study evaluated the mixing performance of human blood at angles of sharp edges of 10°, 15°, 30°, and 45° under the conditions of *Re* = 0.1, *f*_0_ = 5.5 kHz, and *d*_0_ = 5 μm. Figure 17 shows the mixing efficiency of blood at different angles and compares it with that of aqueous solutions. As can be seen, the mixing efficiency of both solutions decreases with increasing angles of sharp edges. However, due to the higher dynamic viscosity of blood at low *Re*, its mixing efficiency is generally lower than that of aqueous solutions at all angles.

### 3.4. Effect of Applied Frequency

**Figure 18:**
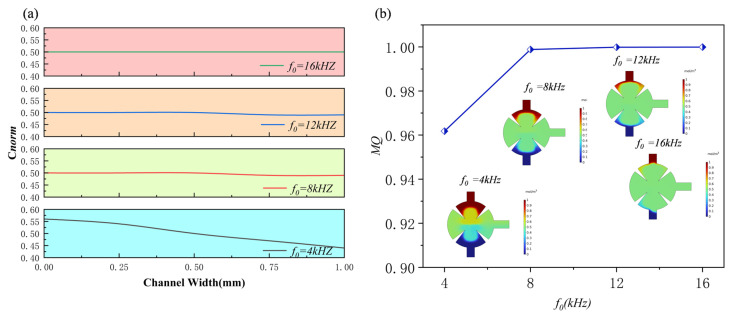
Under different frequencies: (**a**) normalized concentration profile at outlet; (**b**) mixing efficiency profile.

The acoustic frequency strongly influences the mixing performance of the sharp-edge structures acoustically driven micromixer. The intensity and spatial distribution of acoustic streaming are governed by the waves generated at the sharp-edge structures. At lower frequencies (kHz range), the acoustic flow generates large-scale vortices that enhance convective mixing. As frequency increases, diffusion is enhanced because the acoustic wavelength gradually approaches the characteristic dimensions of the microchannel. Consequently, vortices concentrate near the sharp-edge structures and form several small-scale structures. At higher frequencies, stronger acoustic attenuation produces complex three-dimensional flow patterns, which may reduce mixing efficiency. Optimal synergy between convective and diffusive mixing requires precise tuning of the operating frequency. When the frequency exceeds a critical value, effective resonance with the sharp-edge structures becomes difficult, and the influence of acoustic streaming decreases markedly. In addition, high frequencies intensify acoustic attenuation and energy dissipation, further reducing mixing efficiency. Figure 18. presents the concentration distributions and normalized results of the disk micromixer at different frequencies (*d*_0_ = 5 μm, *Re* = 0.1, *α* = 15°) when the solution is water. Despite slight differences in the frequency–concentration curves, the overall effect of frequency on mixing performance is negligible. Specifically, the mixing efficiencies are 0.962, 0.998, 0.999, and 0.999 at acoustic frequencies of 4, 8, 12, and 16 kHz, respectively. The normalized concentration remains close to 0.5 across the outlet cross-section, with minimal fluctuation at all frequencies.

**Figure 19:**
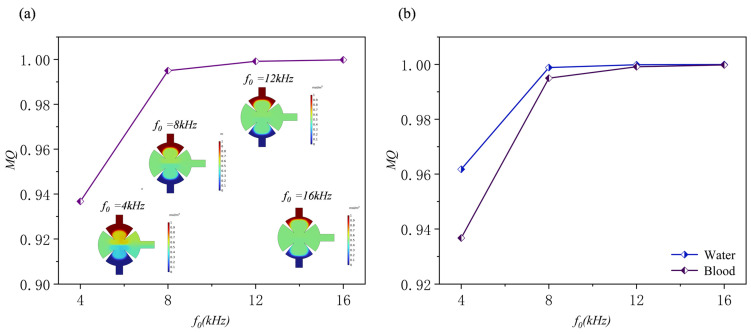
(**a**) Mixing efficiency curves at different frequencies when the fluid is human blood and (**b**) mixing efficiency curves at different frequencies when the fluid is water and human blood.

This study further explored the effect of frequency on mixing efficiency, replacing water with blood to compare mixing performance under different fluid environments. As shown in Figure 19, similar to the case with aqueous solutions, the mixing quality of blood also shows a continuous upward trend with increasing frequency, gradually approaching complete mixing (mixing quality ≈ 1) at higher frequencies. At low frequencies, mixing primarily relies on molecular diffusion; due to the higher viscosity of blood and its weaker diffusion ability, the overall mixing quality in this range is lower than that of aqueous solutions.

## 4. Conclusions

This study investigates an acoustically driven micromixer with sharp-edge structures for biosensing applications. Acoustic streaming, induced by piezoelectric excitation, is analyzed using a disk-shaped mixing chamber. The study systematically explores the effects of acoustic excitation intensity (displacement amplitude *d*_0_), Reynolds number (*Re*), the angles of sharp edges (*α*), and frequency (*f*_0_) on mixing, using a multiphysics framework that couples acoustic streaming with background flow. Results show that increasing displacement amplitude improves mixing efficiency at low *Re*, with diminishing returns at higher *Re* due to background flow interference. The *Re* plays a critical role: at low *Re*, acoustic streaming vortices dominate mixing, while at higher *Re*, inertial forces suppress vortex formation. The angles of sharp edges significantly influence the acoustic streaming, with larger angles of sharp-edges reducing perturbations and preserving cell activity, while smaller angles of sharp-edges improve mixing but may risk cell damage. Frequency has minimal impact on mixing performance. Additionally, comparative simulations between water and blood reveal that the higher viscosity and non-Newtonian characteristics of blood significantly suppress acoustic streaming intensity and vortex strength, leading to lower mixing efficiency compared with water under identical excitation conditions. This mixer shows good application potential in the fields of biology and chemistry. For example, when studying the hemolysis phenomenon of serum samples, appropriately reducing the amplitude and frequency of the sharp-angle structure can improve the low-speed mixing effect, and in the synthesis of silver nanoparticles, by adjusting the Reynolds number and oscillation amplitude, rapid and uniform mixing can be achieved, thereby obtaining nanoparticles with uniform particle size. The proposed acoustically driven sharp-edge micromixer provides a theoretical basis for microscale mixing, enabling comparisons of geometric structures and operating parameters.

## Figures and Tables

**Table 1:** Values of each parameter.

Name	Notation	Worth
Density (water)	*ρ*	997 kg/m^3^
Density (blood)	*ρ*_d_	1060 kg/m^3^
Speed of sound (water)	*c*_0_	1485 m/s
Speed of sound (blood)	*c*_d_	1540 m/s
Kinetic viscosity (water)	*μ*	1 × 10^−3^ Pa·s
Bulk viscosity (water)	*μ_b_*	2.47 × 10^−3^ Pa·s
Bulk viscosity (blood)	*μ_d_*	0.056 Pa·s
Diffusion coefficient	*D*	1 × 10^−9^ m^2^/s
Frequency	*f*	5.5 kHz
Temperature	*T*	296 K

**Figure A1:**
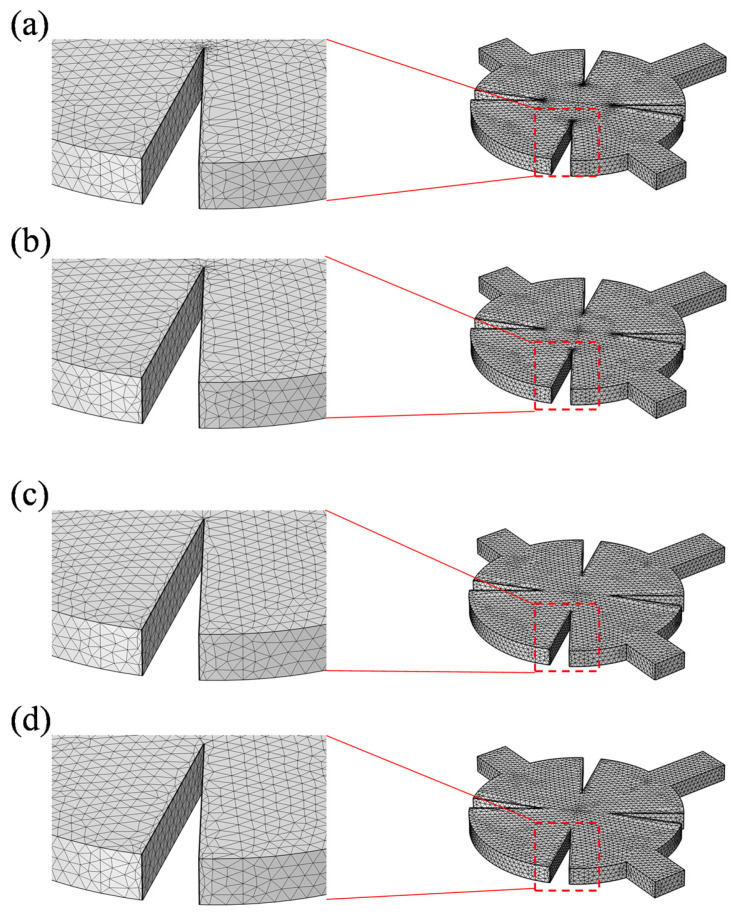
Mesh diagrams at different mesh sizes for various sharp-edge structures: (**a**) maximum cell size: 0.025 mm; (**b**) maximum cell size: 0.05 mm; (**c**) maximum cell size: 0.1 mm; (**d**) maximum cell size: 0.2 mm.

**Figure A2:**
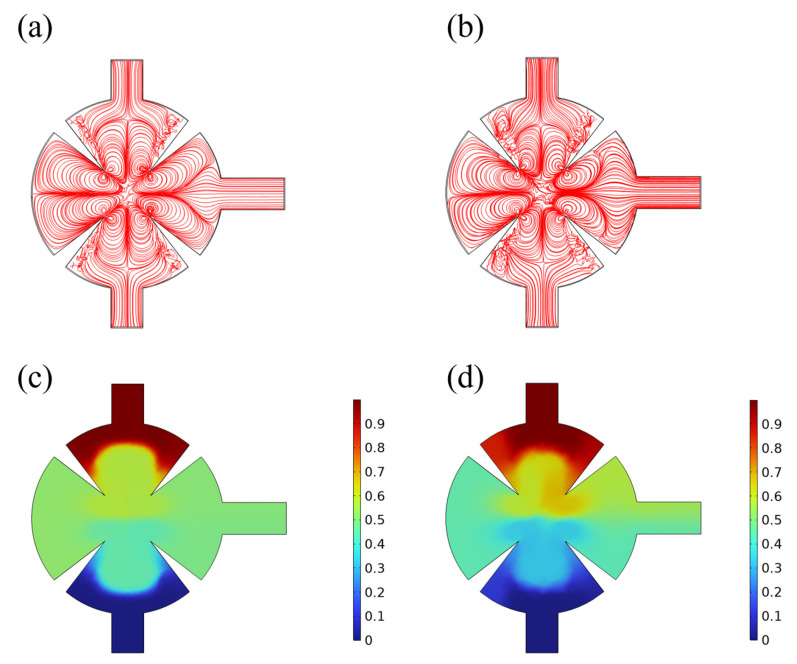
Velocity flow diagram of a sharp-edge structure (**a**) without radius of curvature and (**b**) with radius of curvature. Concentration diagrams for sharp corner structures (**c**) without radius of curvature and (**d**) with radius of curvature.

## Data Availability

Data are contained within the article.

## Appendix A. Grid Independence Test

In this study, we conducted mesh independence verification directly on sharp-edged geometries without applying corner rounding. Sharp-edge structures are critical geometric features for acoustic flow excitation and mixing enhancement. Rounding them would substantially alter the local flow field and deviate from the actual physical structure. The purpose of mesh-independence verification is to confirm the convergence of numerical solutions rather than to smooth locally steep gradients introduced by geometric features. To minimize the influence of angles of sharp-edge singularities on pointwise values, we employed a global performance metric—the outlet mixed concentration index—as the convergence criterion.

Viscous boundary layers form near solid walls, where velocity gradients and shear dissipation serve as the primary sources of steady-state acoustic streaming. When a geometric body is subjected to an acoustic field, the effects of viscous boundary layers on acoustic flow must be accounted for. To achieve reliable acoustic-flow micromixing, the boundary layer must be adequately resolved in the mesh.

The thickness of the acoustic viscous boundary layer is commonly denoted by δ:

(A1) $$\delta =\sqrt{\frac{2\nu }{\omega }}=\sqrt{\frac{\nu }{\pi f}}$$

where ν denotes the kinematic viscosity, set to 1.0 × 10^−6^ m^2^/s in this study, and *ω* = 2*πf*. Three boundary-layer meshes were applied with a stretch factor of 1.15, each layer having a thickness of *δ*. For the internal mixer mesh, the maximum and minimum cell sizes were set to 0.15 mm and 0.01 mm, respectively. In addition, the mesh near sharp-edge structures and the angles of sharp edges were further refined to accurately capture acoustic streaming effects.

The grid study evaluates mesh performance using the outlet mixed concentration as an indicator and quantifies accuracy by the error *ξ* between successive grid results:

(A2) $$\xi =\frac{\left|MQ(h)-MQ(h_{s})\right|}{MQ(h_{s})}$$

where *h* denotes the maximum cell size at the angles of sharp edges and *h_s_* is 0.025 mm in the validation study; cell sizes of 0.025, 0.05, 0.1, and 0.2 mm were tested. The corresponding mesh configurations are presented in Figure A1 and Table A1.

Due to computational constraints, the highest feasible resolution is obtained with a maximum cell size of 0.025 mm at the sharp-edge structures. Mesh independence is considered achieved when the error falls below 2% [33]. Results show that *ξ* varies significantly when *h* decreases from 0.2 to 0.05 mm, but the relative change between *h* = 0.05 mm and *h* = 0.025 mm is less than 2%, indicating convergence at *h* = 0.025 mm. Although a coarser mesh (*h* = 0.2 mm) produces seemingly favorable *ξ* values in some cases, these results arise from discretization errors and numerical dissipation, rather than improved physical accuracy. Taking both convergence and analytical precision into account, *h* = 0.025 is selected as the reference mesh for all subsequent micromixer simulations.

**Table A1:** Number of mesh grids generated by the micromixers.

Maximum Unit Size at the Angles of Sharp-Edge	Number of Cells	Mixed Quality (*MQ*)	Error (*ξ*)
0.025	103,139	0.87441	0
0.05	91,792	0.88636	1.3%
0.1	86,938	0.89957	2.5%
0.2	86,518	0.88335	1%

## Appendix B. Effect of Curvature Radius

The curvature radius of sharp corners is a key geometric parameter that directly affects the intensity of acoustic streaming and, consequently, the mixing performance. When the curvature radius approaches zero, local strain concentration at the sharp-edge structures becomes most pronounced, generating strong acoustic streaming disturbances that enhance mixing efficiency. Increasing the curvature radius weakens this effect. The curvature radius of the sharp-edge structures (r) is typically on the same order of magnitude as the boundary layer thickness (δ). When r >> δ, the corner becomes excessively flat, leading to weak field concentration and limited enhancement of acoustic streaming. When r << δ, such geometry is difficult to achieve both physically and in fabrication. Moreover, the corner becomes much smaller than the effective driving area, which may not yield the highest mixing efficiency. When r ≈ δ or slightly smaller, the acoustic streaming driving force reaches its maximum. At this condition, the geometric scale of the sharp-edge structures matches the physical driving scale, resulting in maximum energy conversion efficiency. Therefore, in this verification, r ≈ 0.5δ (r = 0.0035 mm) was selected to examine the relationship between curvature radius and mixing performance.

In this study, comparisons were conducted under the conditions *d_0_* = 5 μm, *f_0_* = 5.5 kHz, *α* = 15°, and *Re* = 0.1. Figure A2a,b presents streamline patterns for configurations with and without a curvature radius at the sharp-edge structures, while (c) and (d) illustrate the corresponding concentration distributions. The streamline behavior near the sharp-edge structures shows no significant difference between the two configurations, both of which effectively enhance acoustic streaming. Slight variations were observed within the mixing cavity, as a smaller curvature radius produces stronger acoustic driving forces that exert a greater influence on mixing. At the outlet, the mixing quality (*MQ*) values for configurations without and with a curvature radius are 0.99 and 0.95, respectively. These results indicate that both configurations achieve acceptable levels of mixing quality.

This study employed sharp-angle structures without a defined curvature radius to investigate the limiting interaction between acoustic streaming and geometric boundaries, with the goal of revealing the theoretical upper limit of their influence on mixing performance. Although perfectly sharp-edge structures are difficult to achieve in practical fabrication, this idealized model enables the analysis of how curvature radius variations affect acoustic streaming intensity and mixing performance, thereby offering valuable guidance for subsequent structural design.
